# FUME-TCRseq Enables Sensitive and Accurate Sequencing of the T-cell Receptor from Limited Input of Degraded RNA

**DOI:** 10.1158/0008-5472.CAN-23-3340

**Published:** 2024-03-14

**Authors:** Ann-Marie Baker, Gayathri Nageswaran, Pablo Nenclares, Tahel Ronel, Kane Smith, Christopher Kimberley, Miangela M. Laclé, Shreerang Bhide, Kevin J. Harrington, Alan Melcher, Manuel Rodriguez-Justo, Benny Chain, Trevor A. Graham

**Affiliations:** 1 Centre for Evolution and Cancer, Institute of Cancer Research, London, United Kingdom.; 2 Centre for Genomics and Computational Biology, Barts Cancer Institute, Queen Mary University of London, London, United Kingdom.; 3 Division of Infection and Immunity, University College London, London, United Kingdom.; 4 Division of Radiotherapy and Imaging, Institute of Cancer Research, London, United Kingdom.; 5 Department of Pathology, University Medical Center Utrecht, Utrecht, the Netherlands.; 6 Head and Neck Unit, The Royal Marsden Hospital NHS Trust, London, United Kingdom.; 7 Division of Breast Cancer Research, Institute of Cancer Research, London, United Kingdom.; 8 Department of Histopathology, UCL Hospitals NHS Trust, London, United Kingdom.

## Abstract

Genomic analysis of the T-cell receptor (TCR) reveals the strength, breadth, and clonal dynamics of the adaptive immune response to pathogens or cancer. The diversity of the TCR repertoire, however, means that sequencing is technically challenging, particularly for samples with low-quality, degraded nucleic acids. Here, we developed and validated FUME-TCRseq, a robust and sensitive RNA-based TCR sequencing methodology that is suitable for formalin-fixed paraffin-embedded samples and low amounts of input material. FUME-TCRseq incorporates unique molecular identifiers into each molecule of cDNA, allowing correction for sequencing errors and PCR bias. Using RNA extracted from colorectal and head and neck cancers to benchmark the accuracy and sensitivity of FUME-TCRseq against existing methods demonstrated excellent concordance between the datasets. Furthermore, FUME-TCRseq detected more clonotypes than a commercial RNA-based alternative, with shorter library preparation time and significantly lower cost. The high sensitivity and the ability to sequence RNA of poor quality and limited amount enabled quantitative analysis of small numbers of cells from archival tissue sections, which is not possible with other methods. Spatially resolved FUME-TCRseq analysis of colorectal cancers using macrodissected archival samples revealed the shifting T-cell landscapes at the transition to an invasive phenotype and between tumor subclones containing distinct driver alterations. In summary, FUME-TCRseq represents an accurate, sensitive, and low-cost tool for the characterization of T-cell repertoires, particularly in samples with low-quality RNA that have not been accessible using existing methodology.

**Significance:**

FUME-TCRseq is a TCR sequencing methodology that supports sensitive and spatially resolved detection of TCR clones in archival clinical specimens, which can facilitate longitudinal tracking of immune responses through disease course and treatment.

## Introduction

T cells are critical drivers of the adaptive immune response, whose antigen specificity is determined by the highly diverse T-cell receptor (TCR) sequence. High throughput sequencing and analysis of the TCR repertoire have emerged as powerful tools for profiling T-cell responses to pathogens or cancer, or indeed to host tissues in autoimmune disease, however sequencing the TCR locus is particularly challenging. In this manuscript we describe the development, validation, and application of a new TCR sequencing (TCRseq) methodology.

The TCR is a highly diverse heterodimer, which is expressed on the surface of T cells. The majority of T cells express an αβ TCR, whereas γδ T cells generally represent a smaller proportion of the T-cell population. The loci of TCR chains are arranged in segments, with a variable (*V*) region, a diversity (*D*) region (β and δ only), and a joining (*J*) region, followed by a constant (*C*) region. The TCR is produced by a series of stochastic DNA recombination processes, which occur in the early stages of T-cell differentiation in the thymus. During T-cell maturation, one allele of each segment will randomly recombine with the others to form a functional TCR. The β and δ chains undergo VDJ recombination, whereas the α and γ chains undergo VJ recombination. In addition, there is random nucleotide insertion and deletion at the junctions between recombined segments, which generates a huge amount of diversity. Although all undergo recombination, the α, β, γ, and δ chains are in different chromosomal locations and require separate approaches for targeted sequencing. The most variable region of the TCR is the complementarity determining region 3 (CDR3), which is a key component of antigen specificity. The chance of independent generation of the same TCR sequence in a single individual is usually incredibly low, therefore sequencing the CDR3 region of the TCRβ gene can be used as a unique identifier of a T-cell clone. Notably, probabilistic modeling has been used to infer significant differences in the generation probability of specific TCRs (1), and this should be considered when interpreting TCRseq data.

The processes that contribute to the creation of a highly diverse TCR repertoire pose formidable challenges to its genetic analysis. First, because DNA recombination is somatic, the TCRβ locus cannot be predicted from the germline sequence. Second, the stochastic nature of DNA recombination, which results in an enormous amount of genomic diversity means that sequencing across the TCRβ region is particularly challenging. Finally, because clonal amplification of T cells is a fundamental aspect of an immune response, it becomes crucial not only to sequence the TCRs produced in all T cells but to do so quantitatively.

Library preparation for TCRseq can use either genomic DNA (gDNA) or total RNA as input material, and these approaches have been benchmarked (2, 3) and reviewed (4) elsewhere. Protocols that use gDNA have the advantage of a 1:1 relationship between number of cells and sequence abundance, however non-expressed, potentially irrelevant sequences will be detected (4). In contrast, protocols that use RNA have more functional relevance as only expressed transcripts are sequenced, and furthermore they require less input and are more sensitive to rare clonotypes. However, RNA is less stable than DNA, and therefore many commercially available protocols require high-quality RNA as input. Indeed, the majority of existing TCRseq methods are not effective for poor quality input material, such as highly degraded RNA extracted from formalin-fixed paraffin-embedded (FFPE) samples. The few commercial kits that claim to be FFPE-compatible have displayed a poor success rate in our hands and are prohibitively expensive for many researchers. Therefore, we recognized an urgent need for a robust, low-cost, and FFPE-compatible RNA-based TCRseq methodology.

Here we describe our novel protocol, FUME-TCRseq (FFPE-suitable Unique Molecular idEntifier-based TCRseq). The method is multiplex PCR-based, and uses 38 primers against the TCRB V genes. Crucially, it incorporates unique molecular identifiers (UMI) for the correction of amplification bias and sequencing errors. UMIs are essential for quantitative and robust analysis of TCR repertoire data and are not included in many of the current TCRseq methods (particularly those that use gDNA as input material). FUME-TCRseq does not involve template switching or second-strand synthesis; omitting these inefficient steps improves the sensitivity of TCR clonotype detection. Furthermore, FUME-TCRseq targets an amplicon of approximately 170 base pairs, which is shorter than other multiplex methods and enables analysis of highly degraded RNA. FUME-TCRseq requires no specialist equipment and currently costs less than £30 per sample (approximately 10-fold cheaper than commercial equivalents), meaning that it will be accessible to most genomic and molecular biology laboratories.

## Materials and Methods

### Sample collection

The sample of whole blood was collected and sequenced as part of a previously published study (5). The fresh-frozen sample from a patient with inflammatory bowel disease was obtained from St Marks Hospital London, under Research Ethics Committee approval 18/LO/2051, with the patient giving written informed consent. FFPE colorectal cancer samples were collected by the UCLH Cancer Biobank (under Research Ethics Committee approval 15/YH/0311) and the University Medical Center Utrecht. The diagnostic FFPE head and neck cancer biopsies were obtained from The Royal Marsden Hospital, under the INSIGHT-2 study (CCR4934). Institutional board and ethics committee (ref. no. 19/LO/0638) approved the study. This work was conducted in accordance with the provisions of the Declaration of Helsinki.

### RNA extraction

RNA was extracted from the whole blood sample as described previously (5). RNA from macrodissected histologic tissue sections was extracted using the Roche HighPure FFPET RNA Isolation Kit (for FFPE samples) or the AllPrep DNA/RNA Mini Kit (for fresh frozen samples). RNA was quantified using the Qubit 3.0 fluorometer (Thermo Fisher Scientific), and RNA integrity number (RIN) was measured using the Agilent Tapestation 4200.

### RNA/DNA co-extraction from FFPE head and neck tumor samples

Five 10-μm unstained slides and one hematoxylin and eosin-stained slides were obtained from representative FFPE tumor blocks. Experienced pathologists assessed tumor content and suitable areas of tumor were marked for macrodissection, if necessary. RNA and DNA were extracted using the AllPrep DNA/RNA FFPE Kit (Qiagen). Nucleic acid yield and quality were assessed as described above.

### FUME-TCRseq library preparation and sequencing

**Figure 1.**
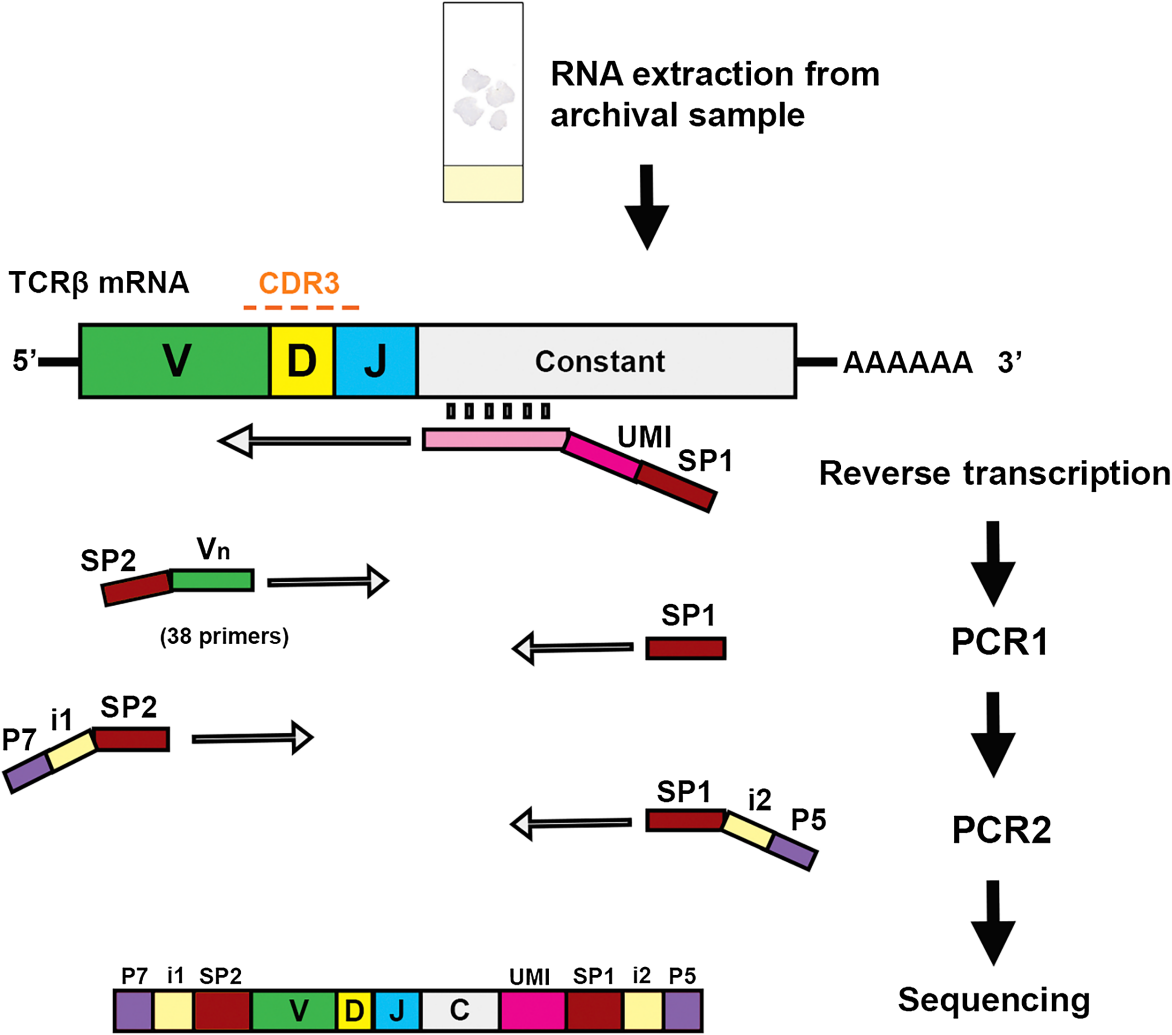
Schematic representation of the FUME-TCRseq methodology, whereby RNA is reverse-transcribed using a UMI-containing primer, followed by multiplex PCR amplification of the CDR3 region using a panel of 38 primers and a second PCR to add sample-specific indexes. SP1 (33bp) and SP2 (24bp) are binding sites for universal Illumina sequencing primers and are used for paired-end sequencing of the amplified TCR and sequencing of the 8bp index 1 (i1) and the 8bp index 2 (i2). P7 (24bp) and P5 (29bp) are flow cell binding sequences for Illumina-sequencing platforms.

A schematic of the FUME-TCRseq protocol is given in Fig. 1, and we provide detailed descriptions of each step below.

#### Step 1. DNase treatment

After quantification, RNA is resuspended in RNase-free water for a final volume of 8 μL. We recommend an input of 50 ng for high-quality RNA (RIN ≥ 7) or 100 ng for samples with low-integrity RNA (RIN < 7). Samples undergo DNase treatment by mixing 8 μL RNA, 1 μL RQ1 DNase (Promega), 1 μL RQ1 10× buffer for 30 minutes at 37°C, then after addition of 1 μL RQ1 DNase stop buffer samples are incubated at 65°C for 10 minutes for inactivation.

#### Step 2. Reverse transcription

First, 6.25 μL of RNase-free water, 1.5 dNTPs (10 mmol/L each), and 0.75 μL “RT oligo” (10 μmol/L, see Supplementary Table S1) are added to the 11 μL DNase-treated RNA, and incubated at 65°C for 5 minutes, before immediately placing on ice for at least 1 minute. Next, 1.5 μL superscript reverse transcriptase (Invitrogen, Thermo Fisher Scientific), 1.5 μL RNasin (Promega), 1.5 μL dithiothreitol (0.1M), and 6 μL 5× Superscript IV buffer are added, and the samples are incubated at 55°C for 20 minutes, then 80°C for 10 minutes.

#### Step 3. Cleanup of cDNA

This purification and all subsequent purification steps are carried out using CleanNGS beads (GC Biotech) according to manufacturer's instructions. The 30 μL reverse transcription product is mixed with 27 μL (0.9×) of CleanNGS beads. After incubation for 5 minutes at room temperature, the beads are collected by placing on a magnetic stand for 2 minutes. The liquid above the beads is carefully aspirated and discarded and the beads are washed twice with 200 μL of freshly prepared 80% ethanol (EtOH), air-dried for 5 minutes and the DNA is eluted in 17.5 μL nuclease-free water. After placing on the magnetic stand for 2 minutes, 16.75 μL of cDNA is transferred to a new tube for PCR1.

#### Step 4. PCR1

A multiplex PCR using the oligo SP1 and a pool of 38 primers designed against the V regions of TCRβ (see Supplementary Table S1) is performed. The V primer pool has been described previously (6). 16.75 μL of cDNA is mixed with dNTPs (0.5 μL, 10 mmol/L stock), SP1 primers (1.25 μL, 10 μmol/L stock), V beta primer pool (1.25 μL), Phusion high fidelity proofreading DNA polymerase (0.25 μL, Thermo Fisher Scientific), and Phusion HF buffer (5 μL, 5 × stock). Initial denaturation was at 98°C for 3 minutes, followed by PCR cycles (98°C 15 seconds, 54°C 30 seconds, 72°C 30 seconds), and final elongation at 72°C for 5 minutes. For high-quality RNA input (from PBMCs or fresh-frozen tissue) 10 cycles of PCR was used, and for low-quality (FFPE samples) 13 cycles was used.

#### Step 5. Cleanup of PCR1

A second purification step using CleanNGS beads at 0.75× (18.75 μL) is performed as previously described in Step 3. The purified product is eluted in 17.5 μL of nuclease-free water, and 16.75 μL is transferred to a new tube for PCR2.

#### Step 6. PCR2

A second PCR is performed to add unique dual indexes. Representative sequences for P5 and P7 primers are shown in Supplementary Table S1. 16.75 μL of purified PCR1 product is mixed with with dNTPs (0.5 μL, 10 mmol/L stock), P5 and P7 primers (1.25 μL each, 10 μmol/L stock), Phusion high-fidelity proofreading DNA polymerase (0.25 μL, Thermo Fisher Scientific), and Phusion HF buffer (5 μL, 5 × stock). For PCR2, initial denaturation was at 98°C for 3 minutes, followed by PCR cycles (98°C 15 seconds, 63°C 30 seconds, and 72°C 40 seconds), and final elongation at 72°C for 5 minutes. For high-quality RNA input, 17 cycles of PCR are used, and for low-quality RNA input, 20 cycles are used.

#### Step 7. Cleanup of PCR2

A final purification step using CleanNGS beads at 0.7× (17.5 μL) is performed before library quantification and sequencing. The final purified product is eluted in 25 μL of nuclease-free water.

#### Step 8. Sequencing

Libraries are quantified using the Qubit fluorometer (Thermo Fisher Scientific), fragment size is assessed using the Agilent Tapestation 4200. Successful libraries yield a single broad peak with fragment size between 350 and 400bp. Libraries are pooled and sequenced on an Illumina MiniSeq or NextSeq (150bp paired end reads) with a 15% PhiX spike-in.

### 5′-RACE library preparation and sequencing

5′-RACE TCRseq libraries were prepared and sequenced as described previously (7).

### Immunoverse TCRseq library preparation and sequencing

We used the Immunoverse-HS TCR Beta/Gamma Kit, for Illumina as per manufacturer's instructions. Libraries were sequenced as described for FUME-TCRseq, with the same target depth.

### ImmunoSEQ library preparation and sequencing

As described previously (8), DNA-based sequencing of the TCRβ chain was done using the ImmunoSEQ Kit (Adaptive Biotechnologies) according to the manufacturer's recommendations. The first round of PCR was carried out using the ImmunoSEQ proprietary PCR primer mix (32 μL per sample containing 25 μL of Qiagen 2× Multiplex PCR Master Mix, 5 μL of Qiagen 5× Q-solution and 2 μL of primer mix). A positive control reaction, provided in the kit, and a negative control reaction were included with each sample batch. PCR cycling parameters were: heated lid (105°C), 95°C 5 minutes denaturation step, followed by 21 cycles of 94°C, 30 seconds denaturation, 65°C, 75 seconds annealing, and 72°C, 40 seconds extension; followed by 72°C, 10 minutes final extension, then hold at 4°C. Amplified libraries were diluted using the DNA suspension buffer (30 μL) provided. A second round of PCR was performed to generate uniquely barcoded sequencing libraries using the barcode primer plate included in the Kit (17 μL of working mix, which include 12.5 μL Qiagen 2× multiplex PCR master mix, 2.5 μL Qiagen 5× Q-solution, and 2 μL of Qiagen RNase-free water; 4 μL of primers from the provided barcode plates and 4 μL of the first PCR product). Second PCR cycling parameters were heated lid (105°C), 95°C 15 minutes denaturation step, followed by 21 cycles of 94°C, 30 seconds denaturation, 68°C, 40 seconds annealing, and 72°C, 60 seconds extension, followed by 72°C, 10 minutes final extension, then hold at 12°C. The quality of the libraries was assessed using Agilent 4200 TapeStation High Sensitivity D1000 ScreenTape. Samples were pooled volumetrically and purified using MAGBIO HighPrep PCR beads (1×). The final pool was quantitated using a Kapa Library Quantification Kit for Illumina. Libraries were sequenced on the Illumina MiSeq System following the manufacturer's instructions and using MiSeq Reagent Kit v3 (150-cycle) Single-Read. A total of 168 sequencing cycles were performed (Read 1 156 cycles, Read 2 12 cycles), as recommended in the protocol, adding 5% PhiX.

### TCR analysis

For FUME-TCRseq, forward and reverse reads were merged using Vsearch (9). Processing of FUME-TCRseq and 5′-RACE-based TCRseq used a previously described suite of Python scripts (7, 10) available at https://github.com/innate2adaptive/Decombinator.

The ImmunoSEQ platform was used for TCR identification and CDR3 extraction of DNA samples sequenced using the ImmunoSEQ Kit (Adaptive Biotechnologies). Further analysis of the TCR repertoires was performed using the Immunarch (11) and VDJtools packages (12).

For libraries sequenced with the Immunoverse-HS TCR beta/gamma Kit, the Archer Analysis platform was used for TCR identification and CDR3 extraction.

### BaseScope point mutation detection

The detection of KRAS G12C and PIK3CA E545K mutations on FFPE sections was performed using the BaseScope assay (Advanced Cell Diagnostics) as described previously (13).

### Data availability statement

The data for this study have been deposited in the European Nucleotide Archive (ENA) at EMBL-EBI under accession number PRJEB72530. All other raw data generated in this study are available upon request from the corresponding author

## Results

### FUME-TCRseq data are consistent with 5′-RACE TCRseq

FUME-TCRseq is a novel protocol, which combines UMI incorporation and enrichment of the TCRβ CDR3 region using multiplex PCR (Fig. 1). We limit our analysis to the hypervariable CDR3 region of TCRβ as it is responsible for the majority of antigen specificity. Our UMIs are 12 random base pairs (two sets of 6 base pairs, separated by an 8 base pair spacer). The addition of UMIs at reverse transcription means that sequencing reads with the same UMI can be traced back to the same initial strand of cDNA. The first advantage of using UMIs is it enables the correction of PCR and sequencing errors. This is particularly important for TCRseq, as TCR repertoires contain nontemplate sequences, which can differ by only one or a few nucleotides from germline sequences and from each other, so it is crucial to determine which sequences represent true T-cell rearrangements and which are artificially generated by errors in PCR or sequencing. Second, the use of UMIs can correct for inherent bias in PCR amplification, allowing more accurate quantification of the relative abundance of each T-cell clone. After reverse transcription we amplify TCRβ CDR3 using a pool of 38 V gene primers (Supplementary Table S1), which were developed and optimized by the EuroClonality-NGS consortium for the monitoring of lymphoid malignancies (6).

**Figure 2.**
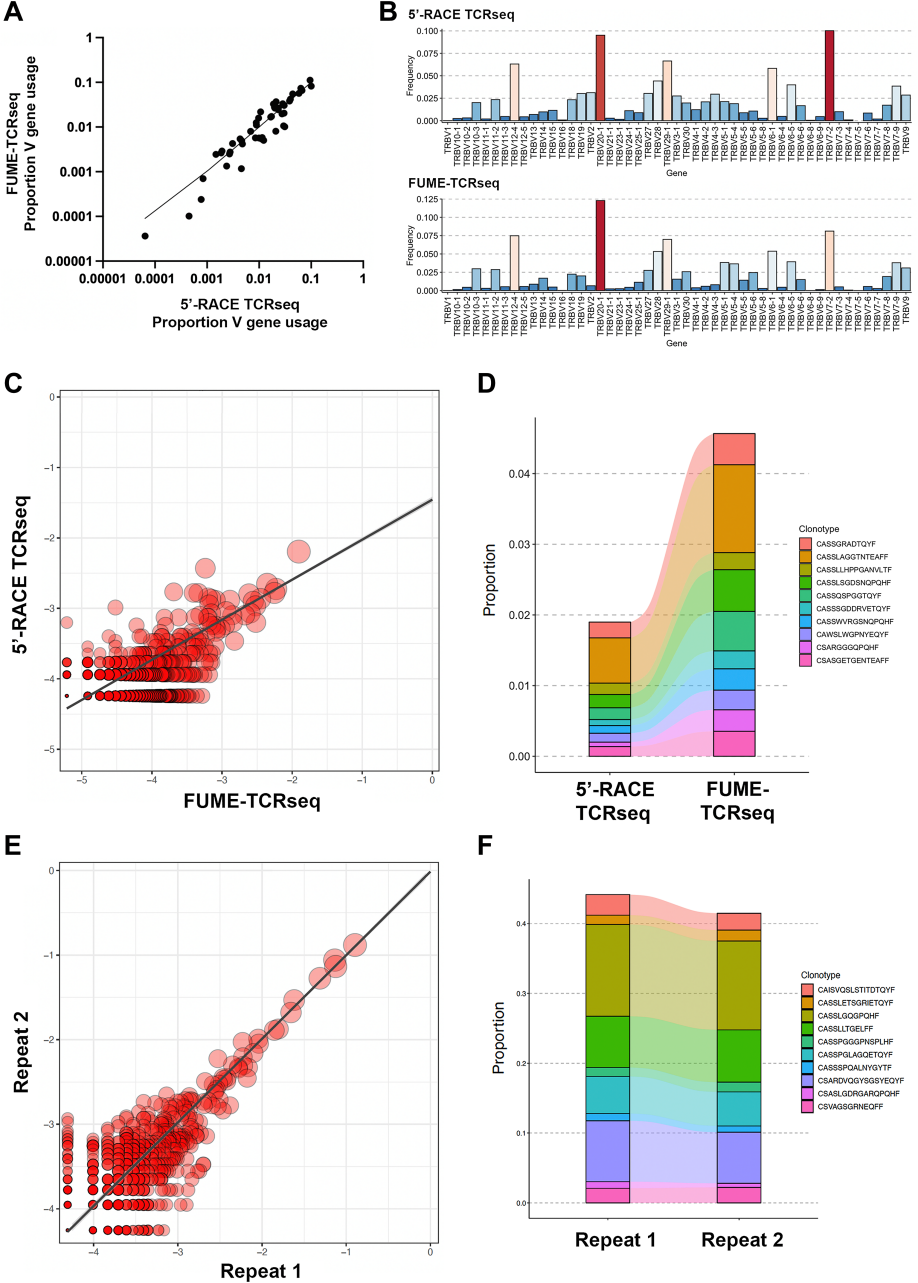
**A,** Scatter plot showing V gene usage data from FUME-TCRseq and 5′-RACE ligation (“5′-RACE TCRseq”) methodologies, performed on RNA extracted from a representative whole blood sample. **B,** Bar plot showing the proportion of each V gene detected by FUME-TCRseq and 5′-RACE ligation. Red bars, highly frequent V genes; blue, less frequent V genes. **C,** Scatterplot showing overlapping clonotype abundances for the FUME-TCRseq and 5′RACE TCRseq data. Point size is scaled to the geometric mean of clonotype frequency in both samples and axes represent log_10_ clonotype frequencies in each sample. **D,** Plot showing the 10 most common clonotypes identified by FUME-TCRseq and the frequencies at which they were detected using 5′-RACE ligation methodology. **E,** Scatterplot showing overlapping clonotype abundances for technical repeats. Point size is scaled to the geometric mean of clonotype frequency in both samples and axes represent log_10_ clonotype frequencies in each sample. **F,** Plot showing the 10 most common clonotypes detected in technical repeats of FUME-TCRseq performed on RNA extracted from an inflammatory polyp.

As a first validation of FUME-TCRseq, we sequenced high-quality RNA extracted from a sample of whole blood. In parallel, we used a well-established 5′-RACE ligation-based method (7) using the same pool of RNA as input (herein called the “5′-RACE method”). Notably, FUME-TCRseq produced a higher proportion of reads mapping to the TCR than the 5′RACE method (98.0% vs. 61.4%, Supplementary Table S2). FUME-TCRseq data were randomly downsampled to the same number of decombined (“mapped”) reads as the 5′RACE data (538942 reads). FUME-TCRseq called many more clonotypes than the 5′-RACE method (113,981 vs. 15,512, Supplementary Table S2), and interestingly had a lower proportion of nonproductive sequences (4.1% vs. 15.5%, Supplementary Table S2). The seven-fold increase in the number of clonotypes called by FUME-TCRseq is likely due to improved efficiency of reverse transcription and amplification, resulting in lower PCR duplication in the sequencing data (average PCR duplication: 3 vs. 22). We found that the data on V gene usage were highly consistent between the two methods (Fig. 2A and B), indicating that the multiplex PCR approach does not skew the data in favor of particular V genes.

We next examined the data for overlap between the specific clonotypes detected. The overlap between the methods was 2,803 clonotypes (18.1% of the 5′-RACE method repertoire, 2.5% of the FUME-TCRseq repertoire, Fig. 2C). However, when we considered only the most expanded clonotypes (those represented by more than 5 UMIs in the 5′-RACE method data), this increased to 74/78 (94.9%). These results indicates a high level of sampling effect in the data. Conversely, the most common clonotypes detected by FUME-TCRseq are also detected by the 5′-RACE method, and at similar relative frequency (Fig. 2D). The Morisita overlap of the FUME-TCRseq and 5′-RACE data were 0.62.

We next extracted high-quality RNA from a fresh-frozen biopsy of a polyp from a patient with inflammatory bowel disease, anticipating a diverse immune repertoire likely with expansions of specific T-cell clonotypes. We tested the reproducibility of FUME-TCRseq by performing duplicate library preparations using the same pool of input RNA. A similar number of unique productive TCR sequences was detected in the replicates (1,787 vs. 2,100). Overall, we detected 34.7% (620/1787) of the clonotypes on repeat (Fig. 2E), but 98.6% (71/72) of the most abundant clones (represented by more than 20 UMIs), reflected in a very high Morisita index of 0.99. Importantly, we detected the most expanded clonotypes in both repeats at very similar proportions (Fig. 2F), confirming good reproducibility of FUME-TCRseq.

### FUME-TCRseq identifies novel T-cell clonotypes in tumor subclones

**Figure 3.**
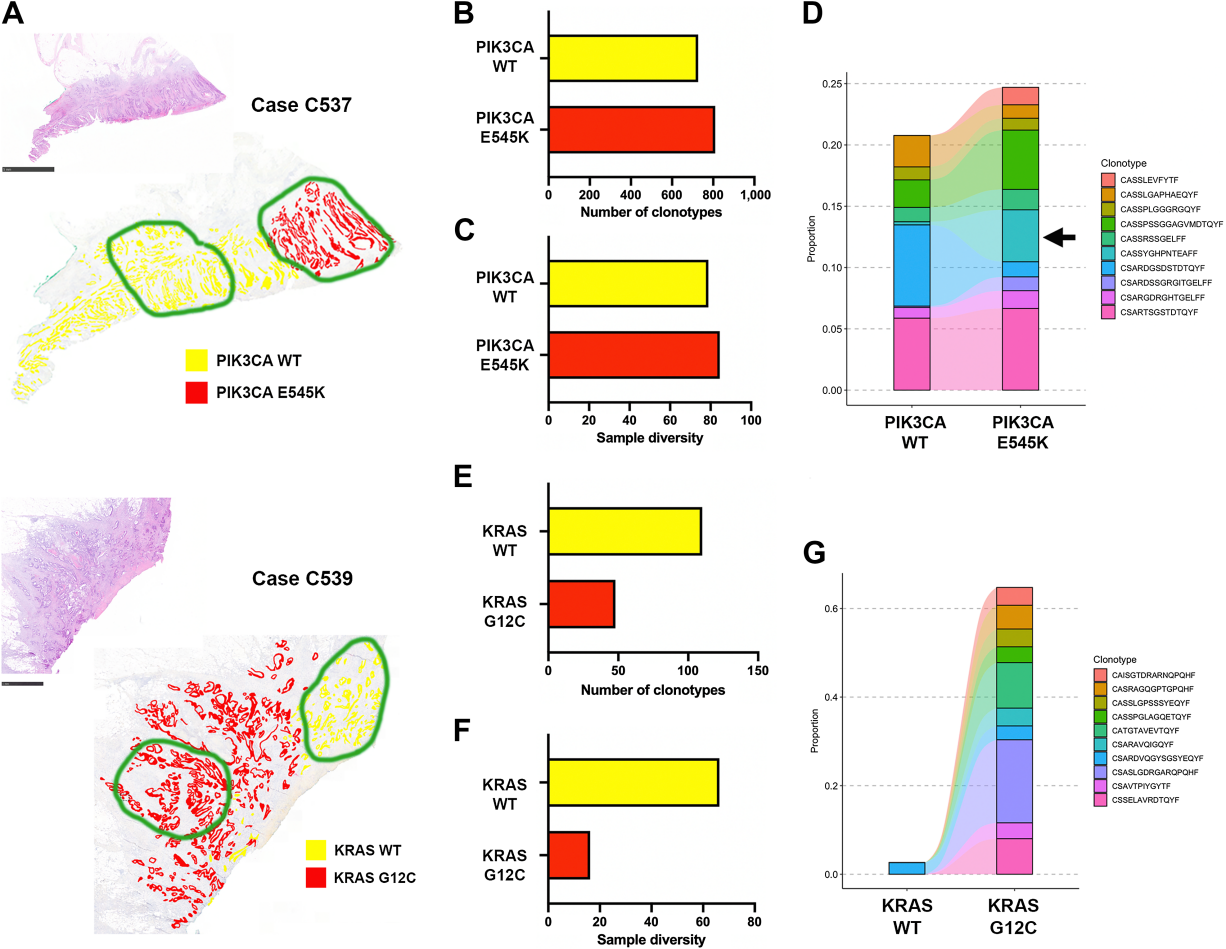
**A,** Hematoxylin and eosin images and annotated BaseScope staining for point mutations in PIK3CA (Case C537, top) or KRAS (Case 539, bottom). Scale bars, 5 mm (top) and 1 mm (bottom). Regions circled in green were macrodissected for FUME-TCRseq. **B,** Bar chart showing the numbers of unique TCR clonotypes detected in the PIK3CA WT and mutant (E545K) subclones of Case C537. **C,** Bar chart showing the diversity of the TCR repertoire in PIK3CA WT and mutant subclones of Case C537. **D,** Plot showing the frequencies of the 10 most common clonotypes detected in the PIK3CA E545K mutant subclone and the frequencies at which they appear in the WT subclone. Arrow, TCR sequence CASSYGHPNTEAFF. **E,** Bar chart showing the numbers of unique TCR clonotypes detected in the KRAS WT and mutant (G12C) subclones of Case C539. **F,** Bar chart showing the diversity of the TCR repertoire in KRAS WT and mutant subclones of Case C539. **G,** Plot showing the frequencies of the 10 most common clonotypes detected in the KRAS G12C mutant subclone and the frequencies at which they appear in the WT subclone.

In our recent multi-omic analysis of primary colorectal cancers, we identified two tumors with subclonal mutations in driver genes (14). We applied the BaseScope assay (point mutation-specific RNA *in situ* hybridization; ref. 13) to an FFPE tissue section to precisely identify cells expressing mutant transcripts (Fig. 3A). Using this annotation as a guide, we macrodissected wild-type (WT) and mutant regions of similar size from serial, unstained FFPE tissue sections of these tumors. We then extracted RNA from these regions and performed FUME-TCRseq. We note that these RNA samples were highly degraded, with RIN of 1.0 (C537) and 2.3 (C539).

In case C537, we found that the PIK3CA WT and E545K-mutant subclones had a similar number of unique TCR clonotypes (727 vs. 810, Fig. 3B; Supplementary Table S2) and similar diversity by the Inverse Simpson index (78.9 vs. 84.5, Fig. 3C). Overall, there was high clonotype overlap between the repertoires (Morisita index 0.64 and Supplementary Fig. S1A). We compared the 10 most common TCR clonotypes in the WT and mutant regions and found that although there were no expanded clonotypes unique to the WT region, there were three clonotypes expanded only in the mutant subclone. Of particular interest is the CDR3 sequence “CASSYGHPNTEAFF,” which represented 3.5% of all TCR reads in the PIK3CA E545K-mutant subclone, and only 0.21% in the PIK3CA WT subclone (Fig. 3D). Furthermore, this CDR3 amino acid sequence was detected six times in the mutant repertoire, with different nucleotide sequences, indicating T-cell convergence towards this clonotype. The spatial distribution of the TCR sequence is suggestive of specificity to a newly generated subclone-specific neoantigen, although we note that this neoantigen is not necessarily the PIK3CA E545K mutation itself.

The subclonal TCR repertoires of case C539 were strikingly different. The KRAS G12C mutant subclone had considerably fewer unique productive TCR clonotypes than the WT subclone (48 vs. 110, Fig. 3E; Supplementary Table S2) and lower diversity by the Inverse Simpson index (16.2 vs. 66.3, Fig. 3F), indicating a differing immune repertoire between the regions. We noted that the WT subclone had 1.7x more sequencing reads than the mutant, therefore we randomly downsampled the WT mapped reads to match that of the mutant and repeated our analysis (Supplementary Table S2). This did not drastically change the number of unique clonotypes (all data = 110, downsampled data = 108) or the diversity (all data = 66.3, downsampled data = 68.8). Clonotype overlap between the repertoires was very low (Morisita index 0.027; Supplementary Fig. S1B). When we examined the ten most expanded clonotypes in the KRAS G12C mutant subclone, only one was present in the KRAS WT subclone (Fig. 3G). We note that factors such as total T cells per region, RNA yield and efficiency of RNA recovery are difficult to experimentally control for in this setting, therefore lack of overlap in TCR repertoires should be interpreted with some caution. Repetition of the experiment should be used to validate findings.

### FUME-TCRseq reveals an altered TCR repertoire at the transition to invasion

**Figure 4.**
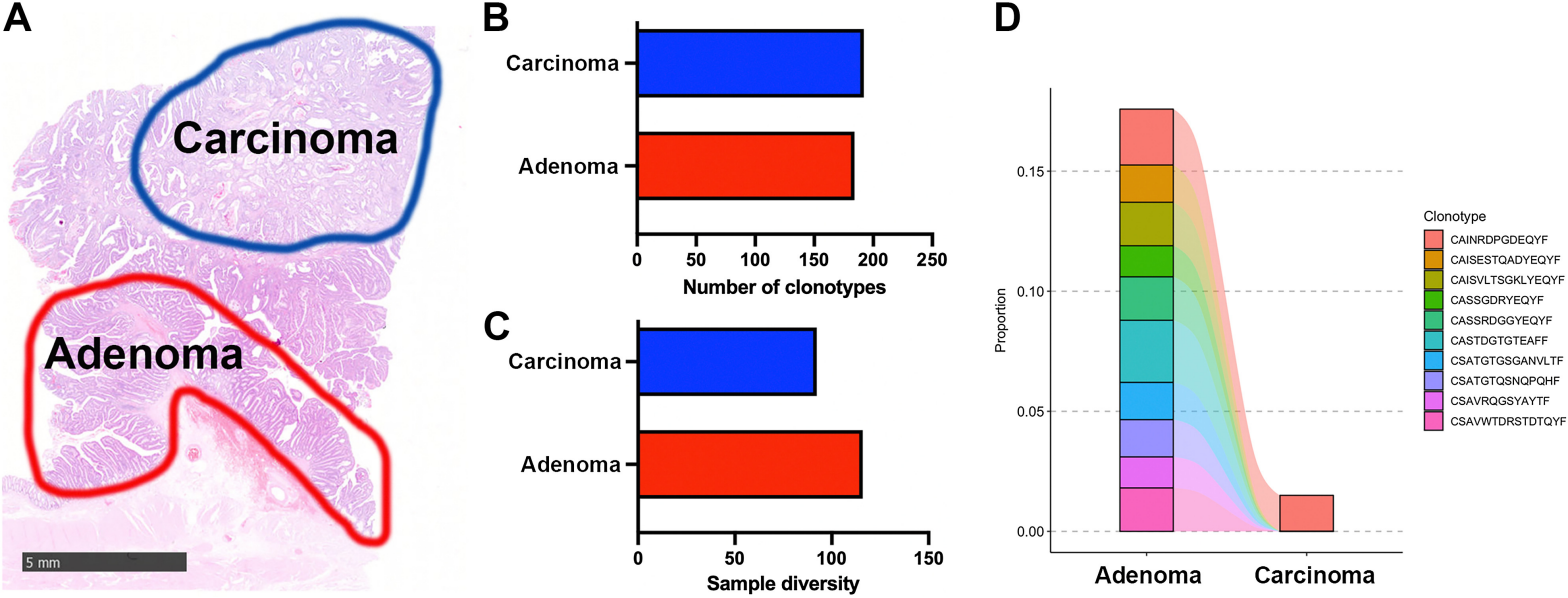
**A,** Hematoxylin and eosin showing the regions of FFPE adenoma and carcinoma that were macrodissected for RNA extraction and FUME-TCRseq. Scale bar, 5 mm. **B,** Bar chart showing the numbers of unique TCR clonotypes detected in carcinoma and adenoma regions. **C,** Bar chart showing the diversity of the TCR repertoire in carcinoma and adenoma regions. **D,** Plot showing the frequencies of the 10 most common clonotypes detected in the adenoma region and the frequencies at which they appear in the carcinoma.

In a recent study we used mathematical modeling combined with sequencing and image-based analysis of the microenvironment to infer the presence of an immune bottleneck at the transition from a benign colorectal adenoma to invasive carcinoma (15). Here we sought to characterize the T-cell repertoires at the invasive transition by performing TCRseq on macrodissected matched adenoma and carcinoma regions (of similar size) from a single FFPE tissue section (Fig. 4A). RNA extracted from these regions had a RIN of 2.3.

We found that the number of unique productive TCR clonotypes detected in adenoma and carcinoma regions was broadly similar (184 vs. 192, Fig. 4B; Supplementary Table S2); however, the carcinoma TCR repertoire was less diverse by the Inverse Simpson index (92.2 vs. 116.0, Fig. 4C). These indicate a broader immune response in the adenoma, with the TCR repertoire of the carcinoma being more focused. Overall, the clonotype overlap was very low (Morisita index, 0.06, Supplementary Fig. S1C), and the regions share only one clonotype in their 10 most expanded clonotypes (Fig. 4D), suggesting a very different T-cell response to cells of the adenoma and carcinoma.

### Comparison of FUME-TCRseq data with industry standards

We compared the performance of FUME-TCRseq against two commercially available methods. First, we chose the Immunoverse assay (ArcherDx), as it uses RNA as input material and is a multiplex PCR-based protocol that incorporates UMIs. To our knowledge it is the most similar protocol to FUME-TCRseq, which is currently commercially available, therefore we considered it to be the most appropriate comparator.

**Figure 5.**
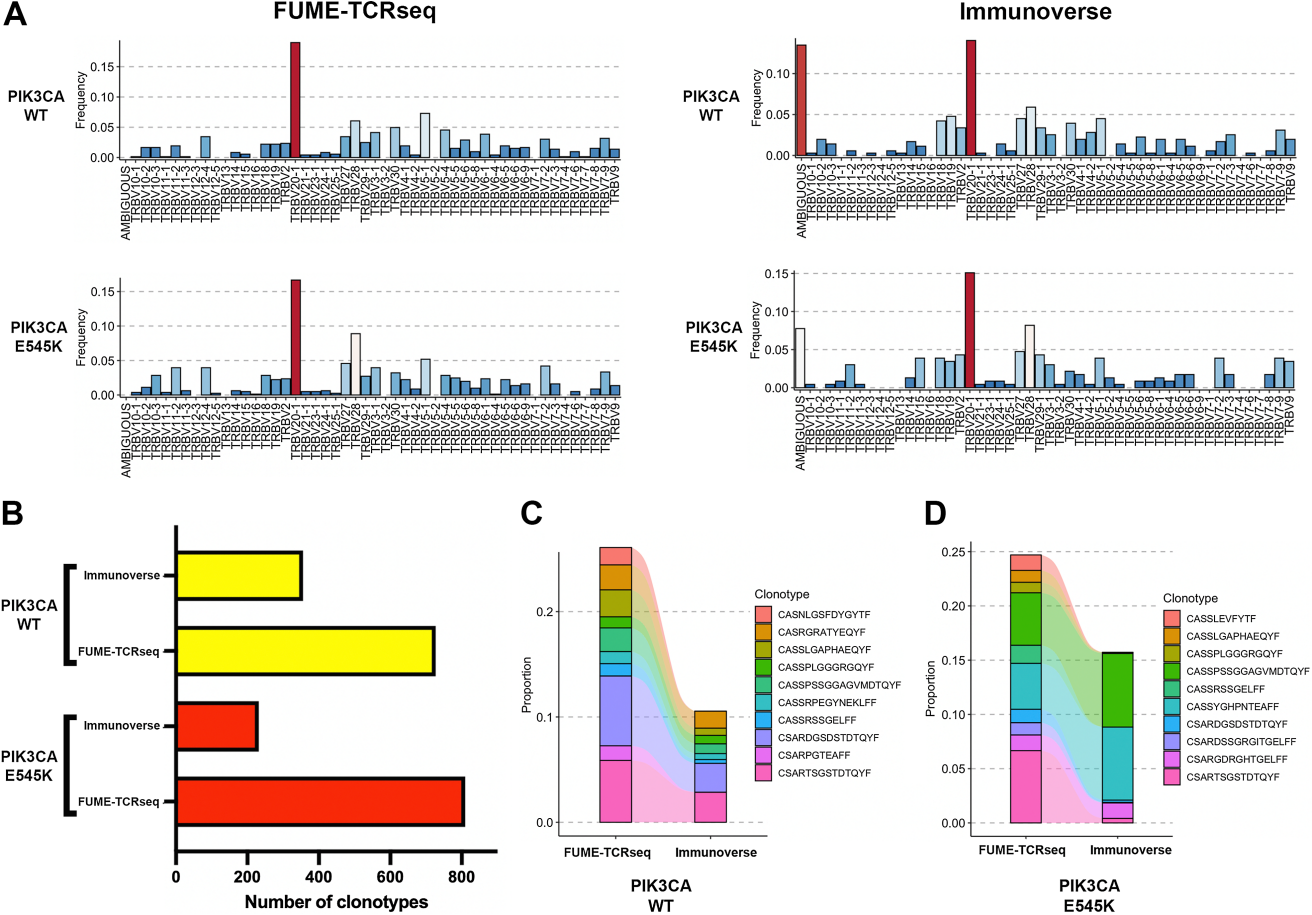
**A,** Bar plots showing the proportion of each V gene detected by FUME-TCRseq (left column) and Immunoverse (right column) in two representative FFPE colorectal cancer samples (PIK3CA WT, top row; PIK3CA E545K, bottom row). **B,** Bar chart showing the numbers of unique TCR clonotypes detected in the two samples with FUME-TCRseq and Immunoverse methodologies. **C,** Plot showing the frequencies of the 10 most common clonotypes detected in the WT sample by FUME-TCRseq and the frequencies at which they appear in the Immunoverse data. **D,** Plot showing the frequencies of the 10 most common clonotypes detected in E545K sample by FUME-TCRseq and the frequencies at which they appear in the Immunoverse data.

We used the WT and mutant samples from C537 (Fig. 3A–D) to compare the methods, running both protocols in parallel with the same amount of input RNA from the same RNA pool. We looked at V gene usage and found it to be broadly consistent between the methods (Fig. 5A), with TRBV20–1 the most frequently used V gene in all cases, representing between 12.7% and 19.7% of all clonotypes. The proportions were lower in the Immunoverse samples, likely because of the large number of “ambiguous” V gene calls.

In both WT and mutant samples, the Immunoverse assay detected fewer unique clonotypes than FUME-TCRseq (Fig. 5B, 356 vs. 727 for WT region, 232 vs. 810 for mutant region; Supplementary Table S2). Although a similar number of sequencing reads per sample was generated for both methods, Immunoverse results had only around 10% on-target reads, whereas FUME-TCRseq had around 70%. This is likely to be a contributing factor to the lower number of clonotypes detected by Immunoverse related to FUME-TCRseq.

We next examined the concordance between the clonotypes detected in the methods. Of 727 clonotypes detected by FUME-TCRseq of sample C537 WT, 105 (14.4%) of these were detected by the Immunoverse assay (Morisita index 0.57, Supplementary Fig. S1D). Considering only the 10 most expanded clones, eight of these were detected by Immunoverse (Fig. 5C). Of the 810 clonotypes detected in FUME-TCRseq of sample C537 mutant, there were 62 (7.7%) detected by Immunoverse (Morisita index 0.57, Supplementary Fig. S1E). Of the 10 most expanded clones detected by FUME-TCRseq, 7 were detected by Immunoverse (Fig. 5D). Interestingly, the previously highlighted clone of interest identified by comparing the WT to mutant regions (“CASSYGHPNTEAFF”) was detected by Immunoverse, and at a similarly high proportion of the total TCR repertoire (6.3% vs. 3.5%).

**Figure 6.**
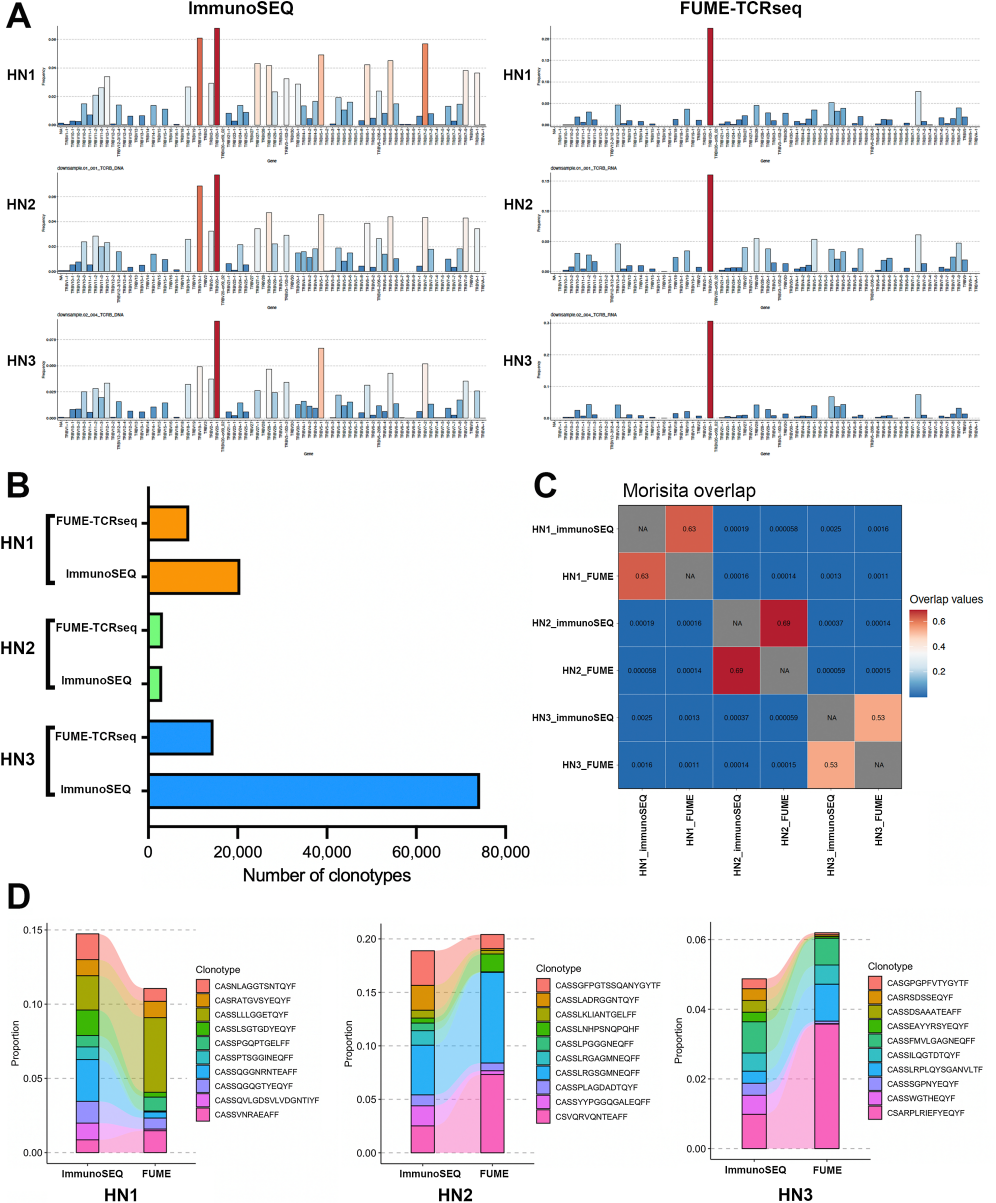
**A,** Bar plots showing the proportion of each V gene detected by FUME-TCRseq (right column) and immunoSEQ (left column) in three representative FFPE head and neck cancer samples (HN1, HN2, HN3). **B,** Bar chart showing the numbers of unique TCR clonotypes detected in the three samples with FUME-TCRseq and immunoSEQ methodologies. **C,** Heat map showing the Morisita overlap between each sample. **D,** Plots showing the frequencies of the 10 most common clonotypes detected in each sample by immunoSEQ and the frequencies at which they appear in the FUME-TCRseq data.

We next compared FUME-TCRseq to the immunoSEQ assay (Adaptive Biotechnologies), which uses gDNA as input material. We extracted DNA and RNA from the same macrodissected tissue isolated from three FFPE head and neck tumors, and used the DNA for immunoSEQ and the RNA for FUME-TCRseq. There were notable differences in V gene usage between the methods (Fig. 6A), which was expected as immunoSEQ detects sequences that are not transcribed and is therefore subject to background amplification of nonfunctional chains. In cases HN1 and HN3, FUME-TCRseq detected fewer clonotypes than immunoSEQ, and in case HN2 it detected slightly more (Fig. 6B; Supplementary Table S2); however, the Morisita overlap between paired immunoSEQ and FUME-TCRseq repertoires was high (Fig. 6C; Supplementary Figs. S1F–S1H). Of all the clonotypes detected by FUME-TCRseq, a mean of 27.2% were detected in the paired immunoSEQ sample. The majority of the most expanded clones detected by ImmunoSEQ were also detected by FUME-TCRseq (Fig. 6D), although relative abundances often varied.

## Discussion

Profiling of the TCR repertoire has wide-ranging applications in health and disease; however, the majority of existing methodologies require large amounts of high-quality input material. Therefore, to date, TCR repertoire analysis of archival FFPE tissue has not been feasible. This manuscript describes FUME-TCRseq, a novel assay for TCR repertoire profiling in highly degraded samples. It is the FFPE-compatibility and error correction via incorporation of UMIs that represent a significant methodological advance over other multiplex PCR-based approaches (16).

We validated the protocol by comparing FUME-TCRseq to a well-established RACE-based TCRseq protocol (7). We found no bias in the detection of V genes, and revealed a high rate of concordance in the detection of specific clonotypes, particularly when only expanded clones were considered. Furthermore, we benchmarked our protocol against a FFPE-compatible commercial kit (Immunoverse, ArcherDX) and found that our method detected a higher number of TCR clonotypes. When comparing our protocol with a commercially available DNA-based TCRseq methodology (ImmunoSEQ), we found that our method detected a smaller number of unique TCRs, most likely due to the fact that DNA-based methodologies tend to overestimate T-cell divergence (2). However, the pair-wise repertoire overlap by the Morisita index was high.

FUME-TCRseq incorporates a UMI on the reverse transcription primer and avoids the inefficient steps of template switching and second-strand synthesis. Similar approaches for sequencing of TCRβ (17) or TCRα (18) have been previously described, however their FFPE compatibility was not evaluated. A proprietary method that utilizes a similar approach of UMI incorporation and multiplex PCR is marketed by MiLaboratories, and although it has been applied to high-quality RNA (19), we are not aware of data that evaluates performance in FFPE material.

The sensitivity of FUME-TCRseq is conducive to novel spatially resolved TCRseq from macrodissected FFPE material, thus enabling multiregion profiling of the T-cell repertoire and correlation with histologic features. We exemplified this by profiling distinct genetic and morphologic subclones of primary colorectal cancers and revealing new insights into immune system co-evolution with tumor cells.

FUME-TCRseq is highly successful in FFPE archival samples, even those with RIN score less than 2. However, we note that FUME-TCRseq library preparation can fail for some samples, and this seems to be largely attributable to particularly low T-cell content. A further limitation of our method is that we only sequence the CDR3 region of the β chain, although this is often considered to be a good surrogate for T-cell clonal identity. Extension of the method to α chain is currently under exploration.

Although we developed FUME-TCRseq for the analysis of highly degraded samples, we have found the protocol to be very robust in the analysis of high-quality RNA samples as well. Therefore, it can be considered a universally applicable method, which is accessible to most researchers due to its low cost and ease of implementation.

In conclusion, FUME-TCRseq is a robust and sensitive novel method for T-cell repertoire profiling, with particular application to highly degraded samples that have hitherto been inaccessible. We anticipate that unlocking the analysis of archival FFPE tissue will facilitate longitudinal analysis of T-cell dynamics in clinical samples, and this has particular relevance in tracking immune responses through disease course and treatment. Broadly, this could reveal novel avenues for biomarker identification and drug development.

## Supplementary Material

Supplementary Data Supplementary Tables 1 and 2, and Supplementary Figure 1

## References

[1] Marcou Q , Mora T, Walczak AM. High-throughput immune repertoire analysis with IGoR. Nat Commun 2018;9:561. 29422654 10.1038/s41467-018-02832-w PMC5805751

[2] Barennes P , Quiniou V, Shugay M, Egorov ES, Davydov AN, Chudakov DM, . Benchmarking of T cell receptor repertoire profiling methods reveals large systematic biases. Nat Biotechnol 2021;39:236–45. 32895550 10.1038/s41587-020-0656-3

[3] Liu X , Zhang W, Zeng X, Zhang R, Du Y, Hong X, . Systematic comparative evaluation of methods for investigating the TCRbeta repertoire. PLoS One 2016;11:e0152464. 27019362 10.1371/journal.pone.0152464 PMC4809601

[4] Nielsen SCA , Boyd SD. Human adaptive immune receptor repertoire analysis-past, present, and future. Immunol Rev 2018;284:9–23. 29944765 10.1111/imr.12667

[5] Ronel T , Harries M, Wicks K, Oakes T, Singleton H, Dearman R, . The clonal structure and dynamics of the human T cell response to an organic chemical hapten. eLife 2021;10:e54747. 33432924 10.7554/eLife.54747 PMC7880692

[6] Bruggemann M , Kotrova M, Knecht H, Bartram J, Boudjogrha M, Bystry V, . Standardized next-generation sequencing of immunoglobulin and T-cell receptor gene recombinations for MRD marker identification in acute lymphoblastic leukaemia; a EuroClonality-NGS validation study. Leukemia 2019;33:2241–53. 31243313 10.1038/s41375-019-0496-7 PMC6756028

[7] Oakes T , Heather JM, Best K, Byng-Maddick R, Husovsky C, Ismail M, . Quantitative characterization of the T cell receptor repertoire of naive and memory subsets using an integrated experimental and computational pipeline which is robust, economical, and versatile. Front Immunol 2017;8:1267. 29075258 10.3389/fimmu.2017.01267 PMC5643411

[8] Nenclares P , Larkeryd A, Manodoro F, Lee JY, Lalondrelle S, Gilbert DC, . T-cell receptor determinants of response to chemoradiation in locally-advanced HPV16-driven malignancies. Front Oncol 2023;13:1296948. 38234396 10.3389/fonc.2023.1296948 PMC10791873

[9] Rognes T , Flouri T, Nichols B, Quince C, Mahe F. VSEARCH: a versatile open source tool for metagenomics. PeerJ 2016;4:e2584. 27781170 10.7717/peerj.2584 PMC5075697

[10] Peacock T , Heather JM, Ronel T, Chain B. Decombinator V4: an improved AIRR compliant-software package for T-cell receptor sequence annotation? Bioinformatics 2021;37:876–8. 32853330 10.1093/bioinformatics/btaa758 PMC8098023

[11] Nazarov V , Tsvetkov V, Rumynskiy E, Popov A, Balashov I, Samokhina M. immunarch: Bioinformatics analysis of T-cell and B-cell immune repertoires. https://immunarch.com/, https://github.com/immunomind/immunarch; 2022.

[12] Shugay M , Bagaev DV, Turchaninova MA, Bolotin DA, Britanova OV, Putintseva EV, . VDJtools: unifying post-analysis of T cell receptor repertoires. PLoS Comput Biol 2015;11:e1004503. 26606115 10.1371/journal.pcbi.1004503 PMC4659587

[13] Baker AM , Graham TA. *In situ* point mutation detection in FFPE colorectal cancers using the BaseScope assay. Methods Mol Biol 2020;2148:349–60. 32394393 10.1007/978-1-0716-0623-0_22

[14] Househam J , Heide T, Cresswell GD, Spiteri I, Kimberley C, Zapata L, . Phenotypic plasticity and genetic control in colorectal cancer evolution. Nature 2022;611:744–53. 36289336 10.1038/s41586-022-05311-x PMC9684078

[15] Gatenbee CD , Baker AM, Schenck RO, Strobl M, West J, Neves MP, . Immunosuppressive niche engineering at the onset of human colorectal cancer. Nat Commun 2022;13:1798. 35379804 10.1038/s41467-022-29027-8 PMC8979971

[16] Montagne JM , Zheng XA, Pinal-Fernandez I, Milisenda JC, Christopher-Stine L, Lloyd TE, . Ultra-efficient sequencing of T cell receptor repertoires reveals shared responses in muscle from patients with myositis. EBioMedicine 2020;59:102972. 32891935 10.1016/j.ebiom.2020.102972 PMC7484536

[17] Ma KY , He C, Wendel BS, Williams CM, Xiao J, Yang H, . Immune repertoire sequencing using molecular identifiers enables accurate clonality discovery and clone size quantification. Front Immunol 2018;9:33. 29467754 10.3389/fimmu.2018.00033 PMC5808239

[18] Komkov A , Miroshnichenkova A, Nugmanov G, Popov A, Pogorelyy M, Zapletalova E, . High-throughput sequencing of T-cell receptor alpha chain clonal rearrangements at the DNA level in lymphoid malignancies. Br J Haematol 2020;188:723–31. 31587259 10.1111/bjh.16230

[19] Zornikova KV , Khmelevskaya A, Sheetikov SA, Kiryukhin DO, Shcherbakova OV, Titov A, . Clonal diversity predicts persistence of SARS-CoV-2 epitope-specific T-cell response. Commun Biol 2022;5:1351. 36494499 10.1038/s42003-022-04250-7 PMC9734123

