# Supplementary material for: FUME-TCRseq Enables Sensitive and Accurate Sequencing of the T-cell Receptor from Limited Input of Degraded RNA
Source: Cancer Res. 2024 Mar 14;84(10):1560–9. doi: 10.1158/0008-5472.CAN-23-3340 (PMC11094417; doi:10.1158/0008-5472.CAN-23-3340)
Supplement: Supplementary Data — Supplementary Tables 1 and 2, and Supplementary Figure 1 [file can-23-3340_supplementary_data_suppsd.pdf]

Supplementary Table 1 – Oligonucleotide details

Oligo name	Sequence (5'-> 3')	Final concentration (μM)
RT oligo	ACACTCTTTCCCTACACGACGCTCTTCCGATCTNNN NNN ATCACGAC NNN NNN ACACAGCGACCTCGGGTGGGAA	0.25
SP1	ACACTCTTTCCCTACACGACGCTCTTCCGATCT	0.5
VB10-2*01	TTC AGA CGT GTG CTC TTC CGA TCTATAAAGGAGAAGTCCCCGATGG	0.025
VB10-3*01	TTC AGA CGT GTG CTC TTC CGA TCTCAAAGGAGAAGTCTCAGATGGC	0.15
VB11-1*01	TTC AGA CGT GTG CTC TTC CGA TCTATCGATTTTCTGCAGAGAGGCT	0.1
VB12-3*01	TTC AGA CGT GTG CTC TTC CGA TCTCCGAGGATCGATTCTCAGCTAA	0.05
VB12-5*01	TTC AGA CGT GTG CTC TTC CGA TCTCTCTAGATGATTCGGGGATGCC	0.025
VB13*01	TTC AGA CGT GTG CTC TTC CGA TCTGGAAGCATCCCTGATCGATTCT	0.0125
VB14*01	TTC AGA CGT GTG CTC TTC CGA TCTCGGTATGCCCAACAATCGATTC	0.1
VB15*10	TTC AGA CGT GTG CTC TTC CGA TCTTGAAGCAGACACCCCTGATAAC	0.025
VB16*01	TTC AGA CGT GTG CTC TTC CGA TCTTCAGCTAAGTGCCTCCCAAATT	0.0125
VB18*01	TTC AGA CGT GTG CTC TTC CGA TCTGCCAAAGGAACGATTTTCTGCT	0.05
VB19*01	TTC AGA CGT GTG CTC TTC CGA TCTCTGAAGGGTACAGCGTCTCTC	0.1
VB2*01	TTC AGA CGT GTG CTC TTC CGA TCTCTCAGTTGAAAGGCCTGATGGA	0.0125
VB20-1*01	TTC AGA CGT GTG CTC TTC CGA TCTTTTCTCATCAACCATGCAAGCC	0.15
VB21-1*01	TTC AGA CGT GTG CTC TTC CGA TCTTGAGCGATTTTTAGCCCAATGC	0.025
VB23-1*01	TTC AGA CGT GTG CTC TTC CGA TCTGGAGATGCACAAGAAGCGATTC	0.15
VB24-1*01	TTC AGA CGT GTG CTC TTC CGA TCTACAAAGGAGAGATCTCTGATGGA	0.025
VB25-1*01	TTC AGA CGT GTG CTC TTC CGA TCTTCCTCTGAGTCAACAGTCTCCA	0.1
VB27-1*01	TTC AGA CGT GTG CTC TTC CGA TCTAGGGAGATGTTCCTGAAGGGTA	0.05
VB28*01	TTC AGA CGT GTG CTC TTC CGA TCTCCTGAGGGGTACAGTGTCTCTA	0.05
VB29-1*01	TTC AGA CGT GTG CTC TTC CGA TCTCTGAGGCCACATATGAGAGTGG	0.1
VB3*01	TTC AGA CGT GTG CTC TTC CGA TCTTTCCCTAATCGATTCTCAGGGC	0.025
VB30*01	TTC AGA CGT GTG CTC TTC CGA TCTCAGAATCTCTCAGCCTCCAGAC	0.05
VB3-1*01	TTC AGA CGT GTG CTC TTC CGA TCTAGTTCCAAATCGCTTCTCACCT	0.025
VB4-1*01	TTC AGA CGT GTG CTC TTC CGA TCTTCGCTTCTCACCTGAATGCCC	0.00625
VB5-1*01	TTC AGA CGT GTG CTC TTC CGA TCTGGAAACTTCCCTGGTCGATTC	0.05
VB5-4*01	TTC AGA CGT GTG CTC TTC CGA TCTGATTCTCAGGTCTCCAGTTCCC	0.15
VB5-5*01	TTC AGA CGT GTG CTC TTC CGA TCTACTTCCCTGATCGATTCTCAGC	0.1
VB5-6*01	TTC AGA CGT GTG CTC TTC CGA TCTCTCAGGTCACCAGTTCCCTAAC	0.1
VB5-8*01	TTC AGA CGT GTG CTC TTC CGA TCTCCTAGATTTTCAGGTCGCCAGT	0.1
VB6-2*01	TTC AGA CGT GTG CTC TTC CGA TCTTACAACTGCCAAAGGAGAGGTC	0.025
VB6-4*01	TTC AGA CGT GTG CTC TTC CGA TCTTACCACTGGCAAAGGAGAAGTC	0.15
VB6-6*01	TTC AGA CGT GTG CTC TTC CGA TCTTAAAGGAGAAGTCCCGAATGGC	0.025
VB6-7*01	TTC AGA CGT GTG CTC TTC CGA TCTGGAGAAGTTCCCAATGGCTACA	0.025
VB7-3*01	TTC AGA CGT GTG CTC TTC CGA TCTCAACGATCGGTTCTTTGCAGTC	0.05
VB7-5*01	TTC AGA CGT GTG CTC TTC CGA TCTTAAATCAGGGCTGCTCAGTGAT	0.05
VB7-7*01	TTC AGA CGT GTG CTC TTC CGA TCTCAGTGATCGGTTCTCTGCAGAG	0.05
VB7-8*01	TTC AGA CGT GTG CTC TTC CGA TCTCTCAACTAGACAAATCGGGGCT	0.1
VB9*01	TTC AGA CGT GTG CTC TTC CGA TCTCTTGAACGATTCTCCGCACAAC	0.05
P5 index*	AATGATACGGCGACCACCGAGATCTACAC**AGCGCTAG**ACACTCTTTCCCTACACGACGCTC	0.5
P7 index*	CAAGCAGAAGACGGCATACGAGAT**CCGCGGTT**GTGACTGGAGTTCAGACGTGTGCTCTTCCGATC	0.5

*Representative P5 and P7 index oligos are given, with the 8 base pair index read in **bold**.

Supplementary Table 2 – Summary of samples and TCR sequencing data

	Type	Figure	RIN	Age of FFPE block (years)	Method	Number of reads input	Number of reads decombined	Number of reads decombined (subsampled)	Total clonotypes	Productive clonotypes	Non productive clonotypes
Blood	FF	2	7.9	N/A	RACE	877926	538942	N/A	18359	15512	2847
Blood	FF	2	7.9	N/A	FUME-TCRseq	1353138	1325926	N/A	143425	137432	5993
Blood_ subsample	FF	2	7.9	N/A	FUME-TCRseq	1353138	1325926	538942	118869	113981	4888
IBD polyp	FF	2	7.8	N/A	FUME-TCRseq	932201	893031	N/A	2273	2100	173
IBD polyp	FF	2	7.8	N/A	FUME-TCRseq	938189	904316	N/A	1936	1787	149
C537 WT	FFPE	3+5	1	3	FUME-TCRseq	922619	594987	N/A	961	727	234
C537 MUT	FFPE	3+5	1	3	FUME-TCRseq	1101308	796627	N/A	1014	810	204
C539 WT	FFPE	3	2.3	3	FUME-TCRseq	37234	32010	N/A	129	110	19
C539 WT_ subsample	FFPE	3	2.3	3	FUME-TCRseq	37234	32010	18482	125	108	17
C539 MUT	FFPE	3	2.3	3	FUME-TCRseq	26053	18482	N/A	58	48	10
Adenoma	FFPE	4	2.3	10	FUME-TCRseq	32224	23039	N/A	233	184	49
Carcinoma	FFPE	4	2.3	10	FUME-TCRseq	36389	22886	N/A	235	192	43
C537 WT	FFPE	5	1	3	Immunoverse	845042	N/A	N/A	478	356	122
C537 MUT	FFPE	5	1	3	Immunoverse	873394	N/A	N/A	317	232	85
HN1	FFPE	6	2.6	2	FUME-TCRseq	117174	93208	N/A	12632	9166	2466
HN2	FFPE	6	2.4	1	FUME-TCRseq	113437	95977	N/A	5805	4530	1275
HN3	FFPE	6	2.6	1	FUME-TCRseq	142133	111457	N/A	18348	14588	3760
HN1	FFPE	6	2.6	2	ImmunoSEQ	unknown	65760	N/A	26862	20630	6232
HN2	FFPE	6	2.4	1	ImmunoSEQ	unknown	6874	N/A	3991	3251	740
HN3	FFPE	6	2.6	1	ImmunoSEQ	unknown	154701	N/A	90070	74297	15773

Supplementary Figure 1

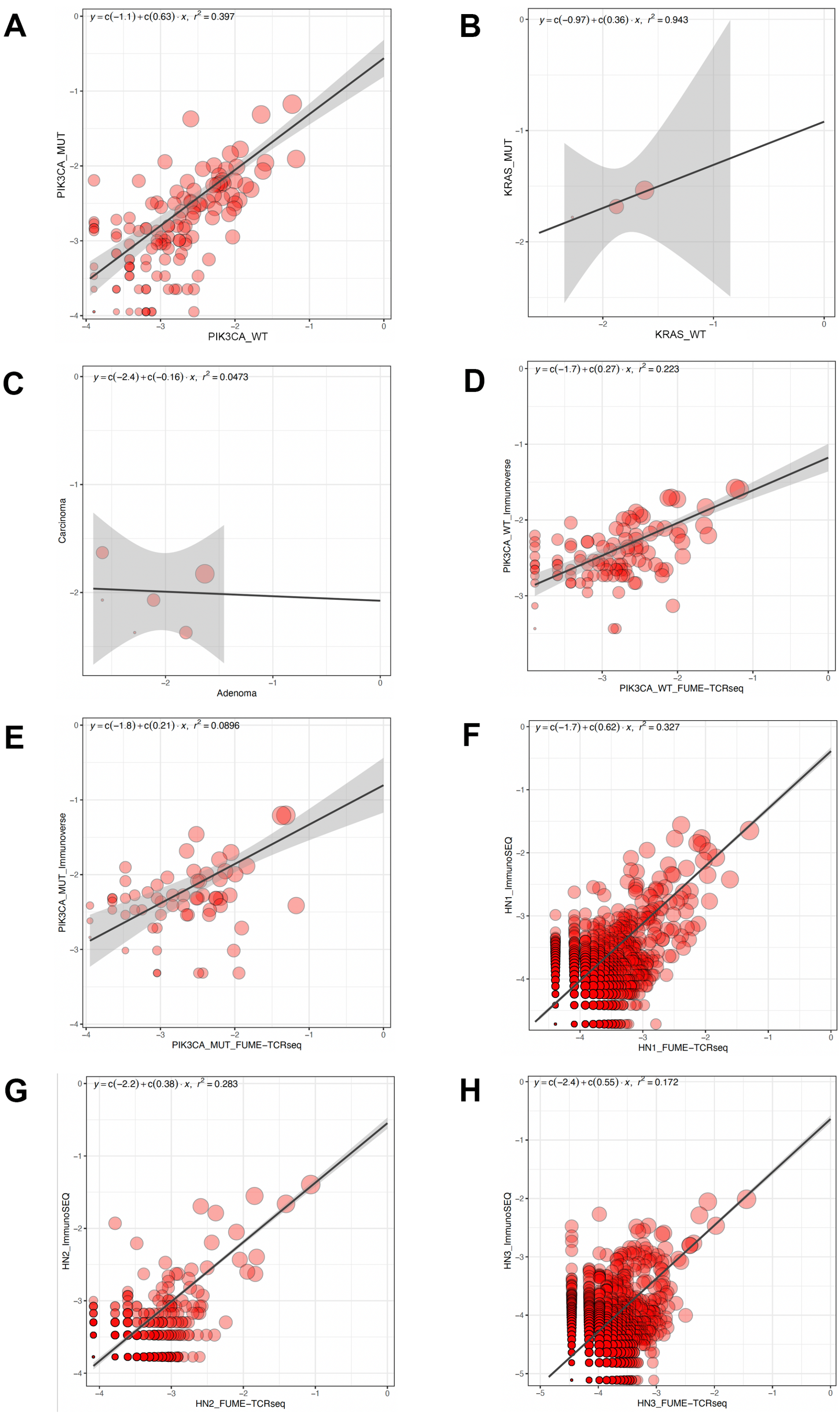

Suplememtary Figure 1

Scatterplots showing pairwise overlapping clonotype abundances for ***(A)*** *PIK3CA WT and mutant subclones,* ***(B)*** *KRAS WT and mutant subclones,* ***(C)*** *matched adenoma and carcinoma,* ***(D, E)*** *FUME-TCRseq vs Immunoverse data,* ***(F, G, H)*** *FUME-TCRseq vs ImmunoSEQ data. Point size is scaled to the geometric mean of clonotype frequency in both samples and axes represent log10 clonotype frequencies in each sample.*
